# Spatial priorities for freshwater biodiversity conservation in light of catchment protection and connectivity in Europe

**DOI:** 10.1371/journal.pone.0267801

**Published:** 2022-05-17

**Authors:** Márton Szabolcs, Felícia Kapusi, Savrina Carrizo, Danijela Markovic, Jörg Freyhof, Núria Cid, Ana Cristina Cardoso, Mathias Scholz, Hans D. Kasperidus, William R. T. Darwall, Szabolcs Lengyel

**Affiliations:** 1 ELKH, Centre for Ecological Research, Institute of Aquatic Ecology, Department of Tisza Research, Debrecen, Hungary; 2 International Union for the Conservation of Nature, Global Species Programme, Freshwater Biodiversity Unit, Cambridge, United Kingdom; 3 Osnabrück University of Applied Sciences, Osnabrück, Germany; 4 German Center for Integrative Biodiversity Research (iDiv), Leipzig, Germany; 5 University of Barcelona, Faculty of Biology, Department of Ecology, Barcelona, Spain; 6 European Commission, Joint Research Centre, Institute for Environment and Sustainability, Water Resources Unit, Ispra, Italy; 7 UFZ − Helmholtz Centre for Environmental Research, Department of Conservation Biology, Leipzig, Germany; University of Florida, UNITED STATES

## Abstract

Freshwater ecosystems host disproportionately high numbers of species relative to their surface area yet are poorly protected globally. We used data on the distribution of 1631 species of aquatic plant, mollusc, odonate and fish in 18,816 river and lake catchments in Europe to establish spatial conservation priorities based on the occurrence of threatened, range-restricted and endemic species using the Marxan systematic conservation planning tool. We found that priorities were highest for rivers and ancient lakes in S Europe, large rivers and lakes in E and N Europe, smaller lakes in NW Europe and karst/limestone areas in the Balkans, S France and central Europe. The *a priori* inclusion of well-protected catchments resulted in geographically more balanced priorities and better coverage of threatened (critically endangered, endangered and vulnerable) species. The *a priori* exclusion of well-protected catchments showed that priority areas that need further conservation interventions are in S and E Europe. We developed three ways to evaluate the correspondence between conservation priority and current protection by assessing whether a cathment has more (or less) priority given its protection level relative to all other catchments. Each method found that priority relative to protection was high in S and E Europe and generally low in NW Europe. The inclusion of hydrological connectivity had little influence on these patterns but decreased the coverage of threatened species, indicating a trade-off between connectivity and conservation of threatened species. Our results suggest that catchments in S and E Europe need urgent conservation attention (protected areas, restoration, management, species protection) in the face of imminent threats such as river regulation, dam construction, hydropower development and climate change. Our study presents continental-scale conservation priorities for freshwater ecosystems in ecologically meaningful planning units and will thus be important in freshwater biodiversity conservation policy and practice, and water management in Europe.

## Introduction

Freshwater ecosystems (rivers, lakes and wetlands) cover less than 1% of the surface of Earth yet harbor 10% of the species described thus far [1]. They provide important ecosystem services such as food production, carbon sequestration, water purification, flood and erosion control [2]. Biological diversity is declining much faster in freshwater ecosystems than in terrestrial and marine ones [3], mainly due to habitat loss, e.g. wetland destruction [4], hydromorphological alterations for flood control and hydropower development [5], habitat fragmentation due to dams and bridges [6, 7], flow alteration [8], impacts of exotic or invasive species [9], pollution [3] and climate change [10, 11]. Yet freshwater ecosystems, habitats and species are still poorly protected globally [12–14] and there is an urgent need for conservation interventions to face these threats [15]. Due to the global and omnipresent nature of threats, the limited resources, and the necessity for interdisciplinary and multi-sectoral approaches to conservation, these interventions need to be prioritized in a scientifically sound manner [16, 17].

Although protected areas are a major foundation of biodiversity conservation, their designation has traditionally been guided by socioeconomic or aesthetic criteria rather than by scientifically sound principles [17]. Protected areas were often designated for terrestrial biodiversity, or, more recently, for marine biodiversity but rarely for freshwater biodiversity [18–20]. Scientifically sound methods such as systematic conservation planning also rarely focus on freshwater ecosystems [16]. Prioritizations for freshwater biodiversity were typically based on river sections or grids as planning units that have little ecological meaning and practical usability, typically on modelled ranges of species of one taxonomic group (most frequently fish) and on the regional spatial scale [21–24]. The uptake of these studies in conservation policy and practice is slow as they are limited by scale and are rarely based on water management units such as catchments [9]. There are few published exercises at larger scales and where catchments are used as planning units [e.g. 25, 26]. In such studies, hydrological connectivity is typically not considered, even though river catchments are inherently connected, which should be accounted for in the prioritization [27, 28].

Conservation planning is also used to evaluate the capacity of existing protected areas to conserve biodiversity [26]. However, few studies investigated the spatial correspondence between hotspots of freshwater biodiversity and protected areas [20, 21, 29]. Similarly, there are only a few prioritizations that consider threat status or degradation [27, 30, 31]. These studies suggest that management should be allocated to catchments that have high levels of biodiversity, are well protected but are vulnerable to future threats, whereas restoration is necessary in catchments that have high levels of biodiversity, are not adequately protected and/or are degraded [22, 32].

The aims of this study were to establish spatial priorities for river and lake catchments in Europe by assessing their importance in the conservation of freshwater biodiversity based on the representation of threatened species, and to evaluate the role of the level of catchment protection (‘catchment protection’ hereafter) and connectivity on conservation priorities. We used an extensive, continental-scale database on the distribution of four ecologically relevant taxa of freshwater ecosystems mapped to catchments as ecologically meaningful planning units in spatial prioritization by the Marxan systematic conservation planning tool. We used selection frequency as a proxy for irreplaceability or the conservation priority of catchments and data on protected areas and connectivity of the catchments to address four questions: (1) Where are the priority areas for the conservation of freshwater biodiversity in Europe? (2) Does the inclusion of well-protected catchments improve the efficiency of prioritization? (3) Where are areas with high biodiversity and low protection, that need further conservation interventions? (4) Is there correspondence between the conservation priority of catchments and their current level of protection? Catchment protection and connectivity are seldom considered in systematic conservation planning exercises for freshwater biodiversity, and such exercises are absent from the Conservation Evidence knowledge hub (http://www.conservationevidence.com). We sought to answer the above questions in three prioritization scenarios in relation to catchment protection, each with and without river connectivity to increase the relevance of the spatial priorities established for conservation policy and practice.

## Materials and methods

We used catchments as planning units, which has several advantages over arbitrary systems of grid cells or hexagons [19, 33, 34]. We used the global HydroBASINS database [35] at level 8 (of 12), where geographical Europe (10 128 044 km^2^) is delineated into 18 816 catchments. Data on the distribution of species were obtained from the IUCN Red List of Threatened Species [36, 37]. Data were from fishes (n = 512 species), molluscs (n = 656), odonates (n = 124), and aquatic plants (n = 339). These groups represent a variety of trophic levels and dispersal types, and are important in ecosystem functioning and services [38–41]. We only used “extant” and “probably extant” occurrences of species in their native range. Three exceptions were the critically endangered molluscs *Belgrandia moitessieri* and *B*. *varica*, which had only “possibly extant” occurrences but were included because of their conservation status, and the critically endangered fish *Scardinius scardafa*, which only has a single introduced population. The final database contained 4,493,267 occurrence records of 1631 species in 18,816 catchments.

We prioritized catchments based on the conservation status, range-restriction and endemism of their species. For conservation status, we used the global Red List status, or, if it was not available (n = 334 species), the European status. Threatened species were those listed as critically endangered (CR), endangered (EN) or vulnerable (VU) [36]. Because CR species are faced with imminent extinction [36], we targeted 100% of their occurrences to be included in the optimized protected area network. For EN and VU species, for which extinction risks are lower [36], we set lower targets (75% of the occurrences for EN species and 50% for VU species) based on [25]. For all other species, at least two occurrences were specified as representation targets.

To estimate range restriction, we calculated range size as the total area of catchments in which the species occurred. We considered a species range-restricted under 20 000 km^2^ for fish and molluscs (taxa with low dispersal ability) and 50 000 km^2^ for odonates and aquatic plants (good dispersal ability) [25]. We targeted 25% of the occurrences of range-restricted species to be included in the optimized network. When a species qualified both as threatened and as range-restricted, conservation status was considered first as it required higher percentages of the species’ occurrences to be included in the optimized network. For non-threatened species whose range was not restricted, we specified two separate occurrences as representation targets.

A species was considered endemic if its range was restricted to one Freshwater Ecoregion of the World [42]. We considered a catchment to hold unique species assemblages if the proportion of ecoregion-restricted fish and mollusc species was at least 5% of all fish and mollusc species [25]. We included the qualifying catchments (n = 190) *a priori* (‘locked in’ by Marxan terminology) into the prioritization.

We used software Marxan for spatial prioritization [43]. Marxan heuristically searches a user-defined number of spatial configurations to identify the one that best meets the user-defined targets of species representation at the lowest cost possible. Marxan was run 1000 times in each scenario studied (see below). In each run, we used the area of planning units as a proxy for the cost of protection [44] to minimize total selected area for efficiency. The maximum total cost of the solutions was specified as 17% of the total area of Europe, corresponding to Target 11 of the global Aichi Biodiversity Targets. We used three Marxan outputs: (i) the network that best met the targets at the lowest cost, (ii) the proportion of species’ ranges covered by the best network, and (iii) the number of times a catchment was selected as part of the optimal network in 1000 runs (selection frequency, a proxy for the irreplaceability of the catchments). We note that “irreplaceability” is used here as a general measure of conservation priority and not in the sense of “ecological irreplaceability” [45].

We obtained spatial data on Natura 2000 protected areas in member states of the European Union (EU) from the European Environmental Agency [46] and on areas protected by national laws from the World Database on Protected Areas (IUCN categories I to IV) [47]. We then calculated the combined proportion of protected areas in each catchment as the sum of all areas protected (either by Natura 2000 or national laws, with the overlap between the two considered only once) divided by catchment area. We used the R statistical environment [48] for all calculations and data preparations and ArcGIS 10.0 for Windows [49] for visualizing the results on maps. All calculations were conducted in the Lambert Azimuthal Equal Area projection (coordinate system GCS_ETRS_1989), which is appropriate for area calculations and is recommended for statistical analysis and display in Europe [50].

To answer Questions 1–3, we ran three scenarios. In Scenario 1, catchment protection was not considered. In Scenario 2, we studied whether the inclusion of well-protected catchments improves the efficiency of prioritization, defined as the number of threatened species represented in the solution given a fixed surface area (17% of the planning area). We implemented this by locking in well-protected catchments *a priori* in the prioritization. We defined a catchment as well-protected if the proportion of protected areas exceeded 70% of the total catchment area. This threshold was chosen to reflect that the macroinvertebrate and fish fauna of pristine and degraded rivers do not differ substantially as long as not more than 30% of the catchment area is transformed to agriculture [51]. The total area of well-protected catchments (n = 1011) was 206,878 km^2^, corresponding to 2% of the total area of geographical Europe. Finally, in Scenario 3, we aimed to identify catchments with high biodiversity and low protection (more than 30% not protected), which may be degraded, and which may thus need further conservation interventions (more protected areas, management, restoration, species protection) by excluding (locking out) well-protected catchments from the prioritization.

To evaluate the effect of catchment connectivity on conservation priorities, we ran each scenario without connectivity and then again with connectivity. In the first case, we used the Boundary Length Modifier (BLM) parameter of Marxan to control for habitat fragmentation (Ball et al. 2009). Based on the results of preparatory Marxan runs (**S1-S3 Figs in S1 File**), we selected an appropriately large value (BLM = 10) to avoid solutions that are too fragmented and to enhance clumping of priority catchments [52]. In the second case, we implemented the idea described by [27] and implemented connectivity in Marxan runs by replacing the BLM file with a river connectivity file that listed catchments and their immediate downstream catchment in a second field. We used the HydroBASINS database to create this file, which contains the outflowing catchments for each river catchment. Because upstream catchments are expected to have a larger influence on catchments downstream than vice versa, we only used upstream connectivity. In Marxan, the parameter Connectivity Penalty (CP) controls the connectivity of the optimal network relative to the area selected for protection. To determine an optimal value for CP, we implemented preparatory Marxan runs with eight different values of CP (**S1 File**). The results suggested that a CP = 10 is a reasonable compromise between costs and connectivity (**S4 Fig in S1 File**).

We developed three ways to assess the correspondence between conservation priority and current level of protection (Question 4) by assuming that if protection is ideal, there should be a strong positive relationship between the proportion of protected areas and irreplaceability. First, we classified catchments into four groups by dividing them into high/low irreplaceability and high/low proportion of protected areas categories at the median values. Second, we calculated residuals from an ordinary least-squares linear regression of irreplaceability as a function of proportion of protected areas. Finally, we measured the deviation of each catchment from an ideal, hypothetical 1:1000 line that is expected if the correspondence between proportion of protected areas (range: 0 to 1) and irreplaceability (range: 0 to 1000) is perfect. In these analyses, we used irreplaceability values obtained in Scenario 1.

## Results

**Fig 1:**
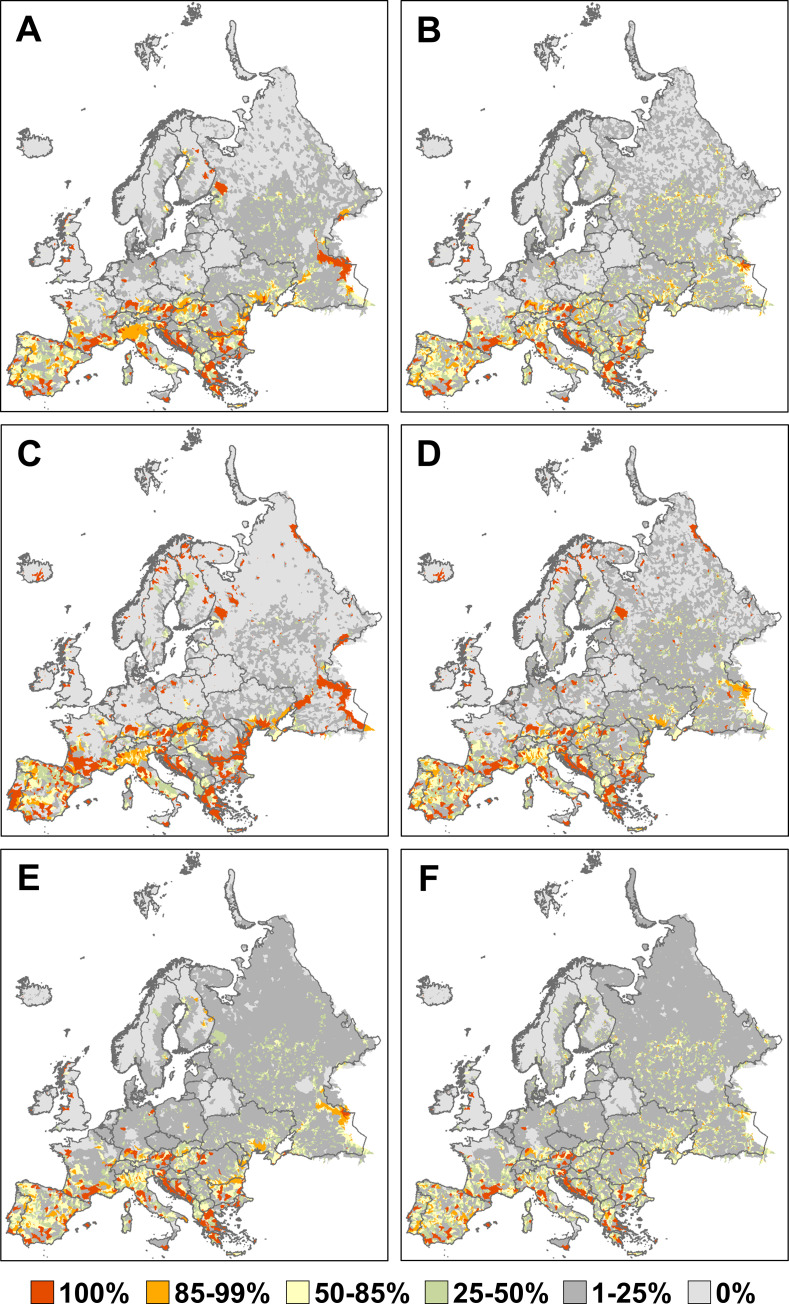
Conservation priority (irreplaceability) of river and lake catchments in Europe (n = 18,816), estimated as selection frequency in 1000 runs of Marxan in three scenarios (rows), each without connectivity (left) or with connectivity (right). In Scenario 1 (upper row), catchment protection was not considered, whereas well-protected catchments (more than 70% of catchment area protected) were *a priori* included in Scenario 2 (middle row) and were *a priori* excluded in Scenario 3 (bottom row). The spatial data for species distributions are deposited in http://project.freshwaterbiodiversity.eu, while the shapefile containing freshwater catchments are available at http://hydrosheds.org/page/hydrobasins. Spatial information on protected areas were obtained from http://protectedplanet.net and http://www.eea.europa.eu. The maps were created with ArcGIS 10.0 by ESRI.

Scenario 1 showed that catchments with high irreplaceability were in S Europe (S Spain, S France, W, S and E Balkans), along major rivers (upper and lower Danube, lower Don, Dniester and Volga), and around large or smaller lakes in N Europe (**Fig 1A**). A list of catchments or catchment groups with a selection frequency of 100% based on Scenarios 1 and 3 along with the country or countries in which they are located is given in (**S1 Table in S1 File**). The proportion of threatened (CR+EN+VU) species for which representation targets were met was 96.3% (547 of 568 species) in Scenario 1 (**Table 1**). When connectivity was considered in the analysis, the variation in irreplaceability increased and high-irreplaceability catchments were less clumped as many smaller river catchments had higher irreplaceability and lake catchments (e.g. Lake Ladoga, lakes in E Finland) had lower irreplaceability (**Fig 1B**). With connectivity, the proportion of threatened species with targets met decreased to 92.6% (526 of 568 species) (**Table 1**).

**Table 1:** Number of species of different Red List conservation status for which representation targets were met (Yes) or not met (No) in the three scenarios, each replicated without or with connectivity.

Red List status	Scenario 1				Scenario 2				Scenario 3			
	Connectivity				Connectivity				Connectivity			
	Without		With		Without		With		Without		With	
	Yes	No	Yes	No	Yes	No	Yes	No	Yes	No	Yes	No
CR	144	9	137	16	147	6	135	18	126	27	107	46
EN	142	5	131	16	144	3	124	23	129	18	111	36
VU	261	7	258	10	262	6	254	14	252	16	232	36
NT	127	0	126	1	127	0	126	1	126	1	126	1
LC	831	3	831	3	832	2	830	4	829	5	828	6
DD	100	2	100	2	100	2	100	2	100	2	99	3
Total	1605	26	1583	48	1612	19	1569	62	1562	69	1503	128

When well-protected catchments were locked in (Scenario 2), irreplaceability increased for catchments in N and central Europe (e.g. in Finland, Iceland, Norway, Poland, Russia, Sweden) and parts of southern Europe (e.g. Portugal) (**Fig 1C**), even when connectivity was considered (**Fig 1D**). The proportion of threatened species with targets met was 97.4% (553 of 568 species) without connectivity and 90.3% (513 of 568 species) with connectivity (**Table 1**).

When well-protected catchments were locked out (Scenario 3), the number of catchments with high irreplaceability decreased, particularly in N and central Europe and along major river systems, but remained high in the W and S Balkans, S Spain, S France and N Alps (**Fig 1E, S1 Table in S1 File**). The addition of connectivity further decreased the number of high-priority catchments and increased the number of catchments with intermediate level of irreplaceability (**Fig 1F**). The proportion of species with targets met was the lowest in this scenario (89.3%, 507 of 568 species) and decreased further when connectivity was considered (79.2%, 450 of 568 species) (**Table 1**).

**Fig 2:**
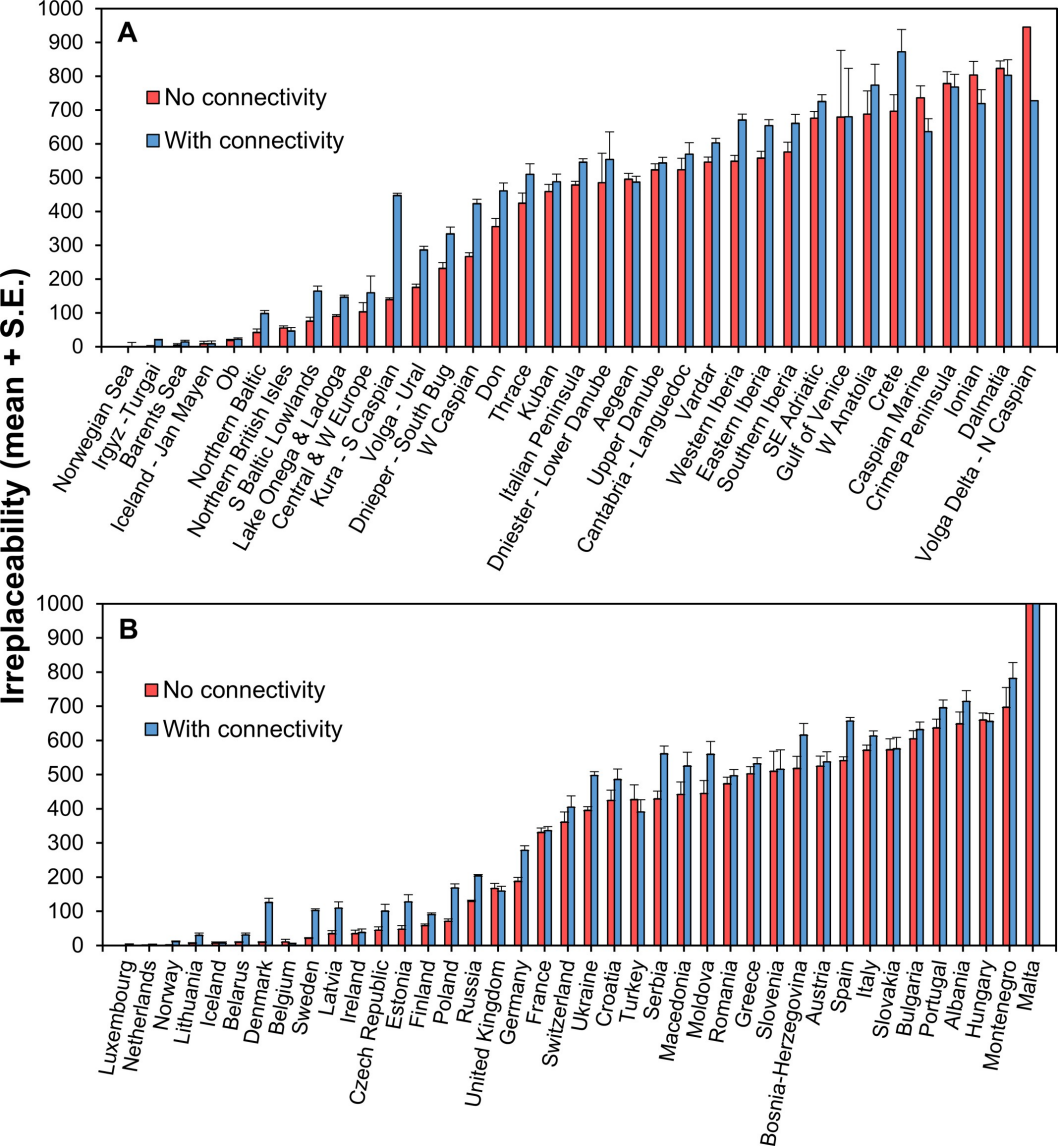
Mean ± SE irreplaceability (selection frequency) of freshwater ecoregions (A) and countries (B) of Europe.

Freshwater ecoregions with the highest average irreplaceability were in S and E Europe, including the catchments of the Volga delta and the N Caspian Sea, the Dalmatian and Ionian coasts, Crimea, the Caspian Sea, Crete, western Anatolia, the N and S Adriatic Sea, followed by southern Iberian and other W Mediterranean ecoregions (**Fig 2A, S1 Table in S1 File**). Adding connectivity to the analysis changed this order slightly but did not affect the importance of the E and the W Mediterranean regions (**Fig 2A**). Accordingly, countries with the highest average irreplaceability also were mostly in S Europe (Malta, Montenegro, Albania, Portugal, Bulgaria, Italy, Spain) and central Europe (Hungary, Slovakia, Austria) (**Fig 2B, S1 Table in S1 File**).The addition of connectivity led to a lower rank for Hungary and the replacement of Austria by Bosnia-Herzegovina in the top 10 (**Fig 2B**).

**Fig 3:**
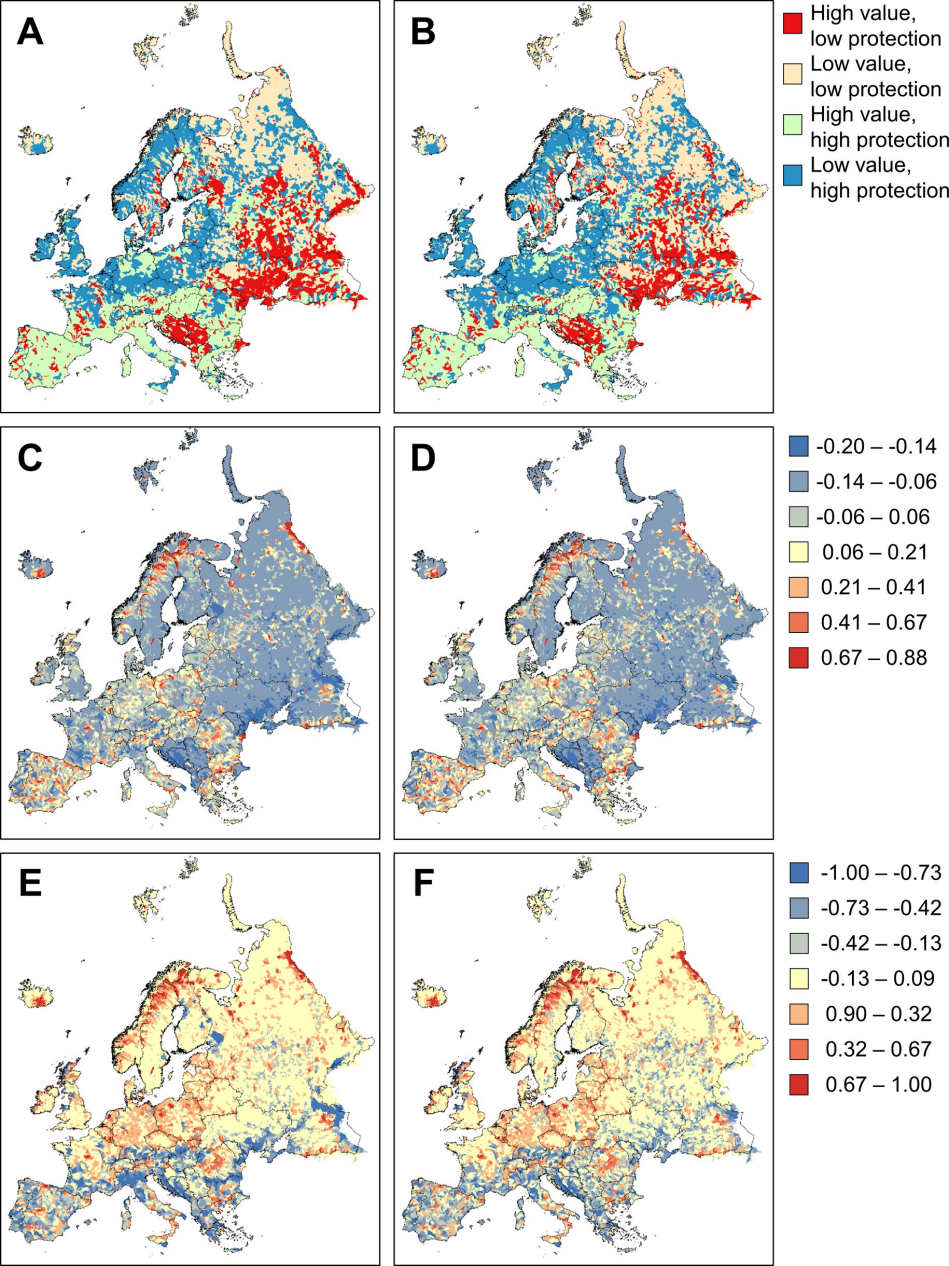
Correspondence between the irreplaceability and the proportion of protected areas per catchment by three methods (rows), each without connectivity (left) or with connectivity (right): distribution of catchments in four combinations of irreplaceability and proportion of protected areas, divided at the medians (A-B); residuals from an ordinary least squares linear regression of irreplaceability over the proportion of protected areas (C-D); and deviations from a hypothetical 1:1000 ideal relationship between proportion of protected areas (range 0 to 1, X axis) and irreplaceability (0 to 1000, Y axis) (E-F). The spatial data for species distributions are deposited in http://project.freshwaterbiodiversity.eu, while the shapefile containing freshwater catchments are available at http://hydrosheds.org/page/hydrobasins. Spatial information on protected areas were obtained from http://protectedplanet.net and http://www.eea.europa.eu. The maps were created with ArcGIS 10.0 by ESRI.

Analyses of the correspondence between the irreplaceability and the proportion of protected areas of catchments showed that catchments in the Balkans (countries of the former Yugoslavia: Bosnia and Herzegovina, Croatia, Montenegro, North Macedonia, Serbia; Albania; Turkish Thrace), in S Ukraine and in central and S Russia had high irreplaceability and low protection (**Fig 3A and 3B**). In contrast, catchments in S Europe generally had high irreplaceability and high protection, those in NW Europe had low irreplaceability and high protection, whereas catchments in NE Europe had low irreplaceability and low protection (**Fig 3A and 3B**).

There was a weak positive relationship between the proportion of protected areas and irreplaceability (no connectivity: slope 170.7 ± 10.11; r = 0.122; n = 18,816; p < 0.0001; with connectivity: slope 217.1 ± 11.23; r = 0.140; n = 18,816; p < 0.0001). The residuals from these regressions confirmed that catchments in the W Balkans, S Ukraine and S Russia had higher irreplaceability than predicted based protection level (**Fig 3C and 3D**). Catchments with lower irreplaceability than predicted were in N Europe and in central and S Europe (**Fig 3C and 3D**). Catchments on the Iberian peninsula showed a particularly mixed pattern, whereas smaller residuals showed adequately protected catchments in central Europe and the E Balkans but less so in N Italy and SW France (**Fig 3C and 3D**).

Finally, deviations from the ideal 1:1000 line showed high irreplaceability and low protection in most catchments south of the 49° latitude (i.e., Upper Danube), and in the lower Volga and Lake Ladoga (**Fig 3E and 3F**). The majority of catchments N from the 49° latitude did not show large deviations (**Fig 3E and 3F**), indicating more or less adequate protection. In contrast, many catchments in northern Europe showed negative deviations, i.e., lower irreplaceability than expected based on protection (**Fig 3E and 3F**), with large deviations especially in N Europe and smaller deviations in central Europe. The addition of connectivity did not influence these patterns in any of the three analyses of correspondence (**Fig 3B–3D and 3F**).

## Discussion

Our study represents a first attempt at continental-scale conservation prioritization based on a large database of freshwater biodiversity and catchments as planning units, which simultaneously considers the conservation status, range-restriction of the species, and the uniqueness (endemicity) of species assemblages, and which also evaluates the effects of current protection and hydrological connectivity of catchments. Our results showed high conservation priority of rivers and lakes in S Europe, large rivers in E Europe and lakes in N Europe, and of karst/limestone areas in the W Balkans and NW Greece, S France, W Bavaria and the E Alps (**Fig 1**). Freshwater ecoregions and countries with the highest average catchment irreplaceability were in S, E and central Europe, with the E Mediterranean and south-central ecoregions ranking slightly higher than ecoregions in the W Mediterranean.

Hydrological connectivity had little influence on the spatial distribution of priorities, although irreplaceability values were more balanced throughout Europe and showed higher averages in ecoregions and countries when connectivity was included than when it was not (**Fig 2**). More importantly, however, the number of threatened species for which representation targets were not met doubled or tripled with connectivity compared to without connectivity in each of the three scenarios (**Table 1**). This result draws attention to a possible trade-off between the inclusion of connectivity and meeting species representation targets [53]. In practice, this trade-off means that the inclusion of connectivity can compromise the protection of some of the threatened species when the maximum amount of land which can be protected is fixed [54]. Moreover, when the emphasis is on river connectivity in a landscape with different water bodies, lakes might appear as less important. Finally, we found that the inclusion of connectivity did not really affect the correspondence between irreplaceability and current protection, which may suggest that connected rivers *per se* were not a priority in the designation of protected areas in the past in Europe. Although the inclusion of hydrological connectivity of catchments into prioritization with systematic conservation planning for freshwater biodiversity is highly desirable [26], these potential issues should be considered in future systematic conservation planning efforts.

The comparison of conservation priority and current catchment protection showed inadequate protection of many catchments in S Europe and generally high protection of catchments in NW Europe relative to their conservation priority. The main difference was that high-priority catchments in S and E Europe were adequately covered by protected areas in countries with the Natura 2000 network (EU member states) but not in others (former Yugoslavia, Ukraine, S Russia) (**Fig 3**). This result suggests that existing protected areas, in particular, the Natura 2000 network, are probably well placed in Europe for freshwater biodiversity conservation and that they adequately cover the freshwater groups studied here, especially in countries with the Natura 2000 network.

The *a priori* inclusion of well-protected catchments (Scenario 2) also resulted in geographically more balanced priorities (cf. **Fig 1A** vs. **1C**) and a more efficient solution (slightly better coverage of threatened species: 97.4% or 553 species in Scenario 2 vs. 96.3% or 547 species in Scenario 1). This result indicated that the consideration of current protected areas produced a similar or better network than a completely new network found by the algorithm. Such an outcome is not evident as similar studies have often reported mismatches between conservation priority and current protection [45, 55]. It also provides hope that the ongoing designation of Natura 2000 protected areas in countries aspiring to EU membership in the Balkans will also better cover freshwater biodiversity than the existing national networks of protected areas.

The *a priori* exclusion of well-protected catchments (Scenario 3) further refined the results of the analysis of correspondence between priority and protection by providing a more detailed map on priority catchments with less than 70% area protection and increased chances of degradation [51], which may thus need further conservation interventions. These catchments are mostly located in S and E Europe (W and S Balkans, S Spain, S France, N Alps; cf. **Fig 1A** vs. **1E**). Scenario 3 also resulted in the lowest coverage of threatened species (89.3% or 507 species without connectivity, 79.2% or 450 species with connectivity) as it ignored occurrences of threatened species in well-protected catchments, demonstrating that the inclusion of well-protected catchments is essential to achieve species representation targets.

The importance of catchments in S Europe corresponds well with biogeographical patterns and processes. Areas in southern Europe provided refuges during Pleistocene glaciation events, and the post-glacial recolonization of northern areas took place from these refuges [56], mainly from the middle and lower Danube basin in the case of freshwater fish [57, 58] and macroinvertebrates [e.g. 59]. Southern areas separated from northern ones by mountain ranges that funcion as migration barriers (e.g. Pyrenées in Iberia, Alps in Italy, Dinarides in the western Balkans) evolved isolated faunas rich in endemics [57]. Finally, karst/limestone areas rich in dolinas, underground waterflows and springs could serve both as glacial refuges for some species (e.g. fish) and interglacial refuges for cold-tolerant others (e.g. spring snails), particularly for shell-bearing mollusks dependent on CaCO_3_, while the geomorphological complexity of karst/limestone areas also increases the chances of isolation and diversification [60].

Our results provide a basis for a more efficient allocation of conservation resources to the protection of freshwater biodiversity and will thus be of interest to conservation scientists, water management authorities, policy-makers and the general public. For instance, because well-protected catchments made up 2% of the total planning area, Scenario 2 identified the next best 15% of the catchments necessary to meet species representation targets, whereas Scenario 3 identified 17% of catchments that are suitable candidates for increased protection other than the already well-protected catchments. It is also important to note that catchments in the western Balkans (Bosnia and Herzegovina, Croatia, Montenegro) had consistently high irreplaceability and further need of conservation interventions (**Figs 1, 2**) but are also a global hotspot of ongoing or future hydropower development [5].

This study provides novel insights relevant in conservation policy and practice in addition to a previous identification of critical catchments in Europe [17]. This study presents spatial conservation priorities for all catchments in Europe rather than only for critical catchments (that hold threatened species), thus, it exploits the full benefits of complementarity and irreplaceability in spatial prioritization. In addition, this study uses a broader range of species, includes range-restriction as a new criterion, presents summary statistics on conservation priority of ecoregions and countries, and evaluates priorities relative to the current protection and the connectivity of catchments. Finally, this study presents a list of high-priority catchments, based on both Scenario 1 and 3, in (**S1 Table in S1 File**) that may be of relevance in international conservation policy and practice, e.g. in the allocation of resources to freshwater biodiversity conservation, but they may also be of interest to conservation scientists and practitioners as well as water management authorities, managers and other local and regional stakeholders.

Finally, our approach to measuring the correspondence between priority areas and existing catchment protection provides a novel contribution to methodology in conservation planning. In most studies, this correspondence is typically measured by the identification and quantification of overlapping areas between maps of conservation priority on one hand and maps of current protection on the other [e.g. 61, 62]. This approach typically uses a subset of the entire range of priority, e.g. the top 17% of the planning units, and examines its overlap with existing protected areas. In contrast, our approach uses information from all planning units and from the entire ranges of priority and protection, and it evaluates whether a certain catchment has more (or less) priority given its protection level relative to all other catchments. By quantifying the match between priority and protection in three ways (classification of catchments into four groups of high/low priority and protection, calculation of residual priority from a regression of priority over protection, estimation of the deviation from an ideal perfect match between priority and protection), our approach thus avoids arbitrary decisions on priority and protection levels, and potential biases arising from using only the top-priority or the best-protected areas. Finally, it provides bench-marked information on the correspondence between priority and protection in each catchment, which will be of interest for conservation policy and practice.

In conclusion, our results draw attention to the high priority of catchments in southern Europe, particularly in the Balkans, and in eastern Europe, particularly in southern Ukraine and Russia, and to karst/limestone areas in the conservation of freshwater biodiversity of Europe. These results are directly applicable in European, regional and local conservation efforts and provide a basis for potential future refinements. Two such potential refinements include restricting the prioritization to protected areas designated specifically for freshwater biodiversity, and implementing a spatial constraint on the proportion of area that can be selected in each ecoregion or country to distribute conservation effort more evenly across Europe. Similar continental-scale assessments based on ecologically meaningful planning units and data from terrestrial and marine biodiversity can greatly improve the efficiency of the allocation of international conservation effort.

## Supporting information

S1 File (DOC)

## References

[1] Strayer DL, Dudgeon D. Freshwater biodiversity conservation: recent progress and future challenges. J N Am Benthol Soc. 2010;29: 344–358. doi: 10.1899/08-171.1

[2] Postel SL Carpenter S. 1997. Freshwater ecosystem services In: Daily GC, editor. Nature’s Services. Washington DC: Island Press; 1997. pp.195–214.

[3] Dudgeon D, Arhington AH, Gessner MO, Kawabata Z-I, Knowler DJ, Lévêque C, et al. Freshwater biodiversity: importance, threats, status and conservation challenges. Biol Rev. 2006;81: 163–182. doi: 10.1017/S1464793105006950 16336747

[4] Zedler JB, Kercher S. Wetland resources: Status, trends, ecosystem services, and restorability. Annu Rev Env Resour. 2005;30: 39–74. doi: 10.1146/annurev.energy.30.050504.144248

[5] Zarfl C, Lumsdon A, Berlekamp J, Tydecks L, Tockner KA. Global boom in hydropower dam construction. Aquat Sci. 2014;77: 161–170. 10.1007/s00027-014-0377-0.

[6] Ligon FK, Dietrich WE, Trush WJ. Downstream ecological effects of dams. BioScience 1995;45: 183–192. doi: 10.2307/1312557

[7] Málnás K, Polyák L, Prill É, Hegedüs R, Kriska G, Dévai G, et al. Bridges as optical barriers and population disruptors for the mayfly *Palingenia longicauda*: an overlooked threat to freshwater biodiversity? J Insect Conserv. 2011;15: 823–832. doi: 10.1007/s10841-011-9380-0

[8] Vörösmarty CJ, Sahagian D. Anthropogenic disturbance of the terrestrial water cycle. BioScience 2000;50: 753–765. doi: 10.1641/0006-3568(2000)050[0753:adottw]2.0.co;2

[9] Collares-Pereira MJ, Cowx IG. The role of catchment scale environmental management in freshwater fish conservation. Fisheries Manag Ecol. 2004;11: 303–312. doi: 10.1111/j.1365-2400.2004.00392.x

[10] Markovic D, Carrizo S, Freyhof J, Cid N, Lengyel S, Scholz M, et al. Europe’s freshwater biodiversity under climate change: distribution shifts and conservation needs. Divers Distrib. 2014;20: 1097–1107. doi: 10.1111./ddi.12232

[11] American Fisheries Society (AFS). Statement of World Aquatic Scientific Societies on the need to take urgent action against human-caused climate change, based on scientific evidence. 2020; Available from: http://climate.fisheries.org/world-climate-statement

[12] Rodrigues ASL, Andelman SJ, Bakarr MI, Boitani L, Brooks TM, Cowling RM, et al. Effectiveness of the global protected area network in representing species diversity. Nature 2004;428: 640–643. doi: 10.1038/nature02422 15071592

[13] Darwall WRT, Holland RA, Smith KG, Allen D, Brooks EGE, Katarya V, et al. Implications of bias in conservation research and investment for freshwater species. Conserv Lett. 2011;4: 474–482. doi: 10.1111/j.1755-263X.2011.00202.x

[14] Maxwell SL, Cazalis V, Dudley N, Hoffmann M, Rodrigues ASL, Stolton S, et al. Area-based conservation in the twenty-first century. Nature. 2020;586: 217–227. doi: 10.1038/s41586-020-2773-z 33028996

[15] Vörösmarty CJ, McIntyre PB, Gessner MO, Dudgeon D, Prusevich A, Green P, et al. Global threats to human water security and river biodiversity. Nature 2010;467: 555–561. doi: 10.1038/nature09440 20882010

[16] Linke S, Turak E, Nel J. Freshwater conservation planning: the case for systematic approaches. Freshw Biol. 2011;56: 6–20. doi: 10.1111/j.1365-2427.2010.02456.x

[17] Carrizo SF, Lengyel S, Kapusi F, Szabolcs M, Kasperidus HD, Scholz M, et al. Critical catchments for freshwater biodiversity conservation in Europe: identification, prioritisation and gap-analysis. J Appl Ecol. 2007;54: 1209–1218. doi: 10.1111/1365-2664.12842

[18] Saunders DL, Meeuwig JJ, Vincent ACJ. Freshwater protected areas: Strategies for conservation. Conserv Biol. 2002;16: 30–41. doi: 10.1046/j.1523-1739.2002.99562.x 35701954

[19] Abell R, Allan JD, Lehner B. Unlocking the potential of protected areas for freshwaters. Biol Conserv. 2007;134: 48–63. doi: 10.1016/j.biocon.2006.08.017

[20] Herbert ME, McIntyre PB, Doran PJ, Allan JD, Abell R. Terrestrial reserve networks do not adequately represent aquatic ecosystems. Conserv Biol. 2010;24: 1002–1011. doi: 10.1111/j.1523-1739.2010.01460.x 20337671

[21] Abellán P, Sánchez-Fernández D, Velasco J, Millán A. Effectiveness of protected area networks in representing freshwater biodiversity: the case of a Mediterranean river basin (south-eastern Spain). Aquat Conserv. 2008;17: 361–374. doi: 10.1002/aqc.778

[22] Thieme M, Lehner B, Abell R, Hamilton SK, Kellndorfer J, Powell G, et al. Freshwater conservation planning in data-poor areas: An example from a remote Amazonian basin (Madre de Dios River, Peru and Bolivia). Biol Conserv. 2007;135: 484–501. doi: 10.1016/j.biocon.2006.10.054

[23] Esselman PC, Allan JD. Application of species distribution models and conservation planning software to the design of a reserve network for the riverine fishes of northeastern Mesoamerica. Freshw Biol. 2011;56: 71–88, doi: 10.1111/j.1365-2427.2010.02417.x

[24] Dolezsai A, Sály P, Takács P, Hermoso V, Erős T. Restricted by borders: trade-offs in transboundary conservation planning for large river systems. Biodiv Conserv. 2015;24: 1403–1421. doi: 10.1007/s10531-015-0864-1

[25] Holland RA, Darwall WRT, Smith KG. Conservation priorities for freshwater biodiversity: The Key Biodiversity Area approach refined and tested for continental Africa. Biol Conserv. 2012;148: 167–179. doi: 10.1016/j.biocon.2012.01.016

[26] Lawrence DJ, Larson ER, Liemann CAR, Mims MC, Pool TK, Olden JD. National parks as protected areas for U.S. freshwater fish diversity. Conserv Lett. 2011;4: 364–371. doi: 10.1111/j1755-263x-2011-00185-x

[27] Hermoso V, Linke S, Prenda J, Possingham HP. Addressing longitudinal connectivity in the systematic conservation planning of fresh waters. Freshw Biol. 2011;56: 57–70. doi: 10.1111/j.1365-2427.2009.02390.x

[28] Linke S, Kennard MJ, Hermoso V, Olden JD, Stein J, Pusey BJ. Merging connectivity rules and large-scale condition assessment improves conservation adequacy in river systems. J Appl Ecol. 2012b;49: 1036–1045. doi: 10.1111/j.1365-2664.2012.02177.x

[29] Keith P. The part played by protected areas in the conservation of threatened French freshwater fish. Biol Conserv. 2000;92: 265–273. doi: 10.1016/s0006-3207(99)00041-5

[30] Moilanen A, Leathwick JR, Quinn JM. Spatial prioritization of conservation management. Conserv Lett. 2011;4: 383–393. doi: 10.1111/j.1755-263X.2011.00190.x

[31] Linke S, Hermoso V, Thieme M. Preliminary results of a freshwater biodiversity Marxan analysis for the Democratic Republic of Congo. Kinshana: Program to Reinforce the Protected Area Network, Départment Technique & Scientifique ICCN, 2012a.

[32] Nel JL, Reyers B, Roux DJ, Impson ND, Cowling RM. Designing a conservation area network that supports the representation and persistence of freshwater biodiversity. Freshw Biol. 2011;56: 106–124. doi: 10.1111/j.1365-2427.2010.02437.x

[33] Linke S, Norris RH, Pressey RL. Irreplaceability of river networks: towards catchment-based conservation planning. J Appl Ecol. 2008;45: 1486–1495. doi: 10.1111/j.1365-2664.2008.01520.x

[34] Nel JL, Roux DJ, Maree G, Kleynhans CJ, Moolman J, Reyers B, et al. Rivers in peril inside and outside protected areas: a systematic approach to conservation assessment of river ecosystems. Divers Dist. 2007;13: 341–352. doi: 10.1111/j.1472-4642.2006.00308.x

[35] Lehner B. HydroBASINS Version1.b. Global watershed boundaries and sub-basin delineation derived from HydroSHEDS data at 15 second resolution; 2012. Available from: http://hydrosheds.org/page/hydrobasins.

[36] IUCN, 2013. IUCN Red List of Threatened Species (accessed 22 April 2020). Available from: https://www.iucnredlist.org

[37] Biofresh. Freshwater Information Platform, 2013. Available at: http://project.freshwaterbiodiversity.eu/.

[38] McIntyre PB, Liermann CAR, Revenga C. Linking freshwater fishery management to global food security and biodiversity conservation. Proc Nat Acad Sci USA. 2016;113: 12880–12885. doi: 10.1073/pnas.1521540113 27791055 PMC5111672

[39] Vaughn CC. Ecosystem services provided by freshwater mussels. Hydrobiologia 2018;810: 15–27. doi: 10.1007/s10750-017-3139-x

[40] May ML. Odonata: Who they are and what they have done for us lately: Classification and ecosystem services of dragonflies. Insects 2019;10: 62. doi: 10.3390/insects10030062 30823469 PMC6468591

[41] O’Hare M, Aguiar FC, Asaeda T, Bakker ES, Chambers PA, Clayton JS, et al. Plants in aquatic ecosystems: current trends and future directions. Hydrobiologia 2018;812: 1–11. doi: 10.1007/s10750-017-3190-7

[42] Abell R, Thieme ML, Revenga C, Bryer M, Kottelat M, Bogutskaya N, et al. Freshwater ecoregions of the world: A new map of biogeographic units for freshwater biodiversity conservation. BioScience. 2008;58: 403–414. doi: 10.1641/b580507

[43] Ball IR, Possingham HP, Watts M. Marxan and Relatives: Software for Spatial Conservation Prioritization. In: Moilanen A, Wilson KA, Possingham HP, editors. Spatial Conservation Prioritization: Quantitative Methods and Computational Tools. Oxford: University Press Oxford; 2009. pp. 185–195.

[44] Moilanen A, Leathwick J, Elith J. A method for spatial freshwater conservation prioritization. Freshw Biol. 2008;53: 577–592. doi: 10.1111/j.1365-2427.2007.01906.x

[45] Ardron JA, Possingham HP, Klein CJ. Marxan Good Practices Handbook. Vancouver: Pacific Marine Analysis and Research Association; 2008. doi: 10.1136/jnnp.2007.126045

[46] EEA. Natura 2000 End 2013, 2013 Available from: http://www.eea.europa.eu

[47] UNEP-WCMC and IUCN. The World Database on Protected Areas (WDPA), 2013. Available from: http://protecteplanet.net.

[48] R Core Team 2019. R: A language and environment for statistical computing. Vienna: R Foundation for Statistical Computing. Available from: http://www.R-project.org.

[49] ESRI. ArcGIS 10.0, 2010. Available from: https://support.esri.com/en/products/desktop/arcgis-desktop/arcmap/10.

[50] INSPIRE. D2.8.1.1., 2009. INSPIRE Data Specification of Coordinate Reference Systems–Technical Guidelines (accessed 22 April 2020). Available from: https://inspire.ec.europa.eu/id/document/tg/rs

[51] Allan JD. Landscapes and riverscapes: The influence of land use on stream ecosystems. Annu. Rev Ecol Evol Syst. 2004;35: 257–284. doi: 10.1146/annurev.ecolsys.35.120202.110122

[52] Stewart RR, Possingham HP. Efficiency, costs and trade-offs in marine reserve system design. Environmental Modelling and Assessment 2005;10: 203–213. doi: 10.1007/s10666-005-9001-y

[53] Briers RA. Incorporating connectivity into reserve selection procedures. Biol Cons. 2005;103: 77–83. doi: 10.1016/s0006-3207(01)00123-9

[54] Rothley KD, Rae C. Working backwards to move forwards: Graph-based connectivity metrics for reserve network selection. Environ Model Assess 2005;10: 107–113. doi: 10.1007/s10666-005-4697-2

[55] Zupan L, Cabeza M, Maiorani L, Roquet C, Devictor V, Lavergne S, et al. Spatial mismatch of phylogenetic diversity across three vertebrate groups and protected areas in Europe. Divers Distrib. 2014;30(6): 674–685. doi: 10.1111/ddi.12186 24791146 PMC4001079

[56] Hewitt GM. Post-glacial re-colonization of European biota. Biol J Linn Soc. 1999;68: 87–112. doi: 10.1111/j.1095-8312.1999.tb01160.x

[57] Pattern Griffiths D. and process in the ecological biogeography of European freshwater fish. J Anim Ecol. 2006;75: 734–751. doi: 10.1111/j.1365-2656.2006.01094.x 16689956

[58] Reyjol Y, Hugueny B, Pont D, Bianco PG, Beier U, Caiola N, et al. Patterns in species richness and endemism of European freshwater fish. Global Ecol Biogeogr. 2007;16: 65–75. doi: 10.1111/j.1466-8238.2006.00264.x

[59] Bálint M, Málnás K, Nowak C, Geismer J, Váncsa É, Polyák L, et al. Species history masks the effects of human-induced range loss—unexpected genetic diversity in the endangered giant mayfly *Palingenia longicauda*. PLoS ONE 2012;7: e31872 doi: 10.1371/journal.pone.0031872 22412844 PMC3297596

[60] Oikonomou A, Leprieur F, Leonardos ID. Biogeography of freshwater fishes of the Balkan Peninsula. Hydrobiologia 2014;738: 205–220. doi: 10.1007/s10750-014-1930-5

[61] Brum FT, Graham CH, Costa GC, Blair Hedges S, Penone C, Radeloff VC, et al. Global priorities for conservation across multiple dimensions of mammalian diversity. Proc Nat Acad Sci USA. 2017;114: 7641–7646. doi: 10.1073/pnas.1706461114 28674013 PMC5530698

[62] Cunningham CA, Crick HQP, Morecroft MD, Thomas CD, Beale CM. Translating area-based conservation pledges into efficient biodiversity protection outcomes. Communications Biology 2021;4: 1043. doi: 10.1038/s42003-021-02590-4 34493796 PMC8423728

