# Supplementary material for: Spatial priorities for freshwater biodiversity conservation in light of catchment protection and connectivity in Europe
Source: PLoS One. 2022 May 17;17(5):e0267801. doi: 10.1371/journal.pone.0267801 (PMC9113586; doi:10.1371/journal.pone.0267801)
Supplement: S1 File — (DOC) [file pone.0267801.s001.doc]

SUPPLEMENTARY MATERIAL

to accompany

“Spatial priorities for freshwater biodiversity conservation
in light of catchment protection and connectivity in Europe”

by

Márton SZABOLCS, Felícia KAPUSI, Savrina CARRIZO, Danijela MARKOVIC, Jörg FREYHOF, Nuria CID, Ana-Cristina CARDOSO, Mathias SCHOLZ, Hans D. KASPERIDUS, William R. T. DARWALL, Szabolcs LENGYEL

**Contents**

Table S1. List of catchments with the highest priority for the conservation of freshwater biodiversity in Europe based on Scenario 1 (**Fig. 1A**) and Scenario 3 (**Fig. 1E**).

Part 1. Determining an optimal value for the Boundary Length Modifier (BLM)

Figure S1. Boundary length as a function of area for calibrating the BLM.

Figure S2. The best networks at different levels of the BLM parameter.

Figure S3. Species coverage at different levels of the BLM parameter.

Part 2. Determining an optimal value for the Connectivity Penalty (CP)

Figure S4. Boundary length as a function of area for calibrating the CP.

**Table S1.** List of catchments or catchment groups with the highest priority for the conservation of freshwater biodiversity in Europe (maximum irreplaceability, Marxan selection frequency=100%) in Scenario 1 with no connectivity (protected areas not considered) (**Fig. 1A**) and in Scenario 3 with no connectivity (well-protected areas *a priori* excluded) (**Fig. 1E**).

| **Country** | **River, river system or lake** | **Scenario 1**  **(Fig. 1A)** | **Scenario 3**  **(Fig. 1E)** |
| --- | --- | --- | --- |
| Albania | upper Drino, Lake Butrint, southern coast | + | partly |
| Albania, Montenegro | Cem/Cijevna | + | + |
| Albania, Greece, North Macedonia | Lake Prespa | + | + |
| Albania, North Macedonia | Lake Ohrid | + | + |
| Austria | Glan, Gurk | + | + |
| Austria | Krems, Traun with tributaries: Ager/ Vöckla, Alm; Lakes Traun, Atter, Mond | + | + |
| Austria | lower Aist | + | + |
| Austria | middle Mur and tributaries: Mürz, Kainach | + | + |
| Austria | Schwechat, Fischa, Russbach, Leitha | + | partly |
| Austria | Ybbs, Erlauf, Melk, Mösel, Pielach | + | partly |
| Austria, Hungary | Lake Neusiedler | + | – |
| Austria, Hungary | upper Raab | + | + |
| Bosnia & Herzegovina | middle Una | + | + |
| Bosnia & Herzegovina | Trebišnjica, Lake Bilećko | + | + |
| Bosnia & Herzegovina | Vrbas, Vrbanja, Usora | + | + |
| Bosnia & Herzegovina, Croatia | Cetina, Neretva | + | + |
| Bulgaria | lower Danube and tributaries: Topolovetz, Archar, Skomlya, Malak Iskar, Vit, Barata, Rusenski Lom, Sazlyaka | + | partly |
| Bulgaria | upper, middle Maritsa and tributaries: Topolnitsa, Chepinska, Vacha, Chepelarska, lower Stryama | + | partly |
| Bulgaria, Romania | Almălău, Lake Bugeac | + | – |
| Bulgaria, Romania | Dobruja region small rivers | + | partly |
| Bulgaria, Turkey | Rezovska, Veleka, Sredetzka, Lake Mandrenska, Hadjiyska | + | partly |
| Croatia | Adriatic islands e.g. Brac, Hvar | + | + |
| Croatia | Dobra, Mrežnica, Korana, Plitvice Lakes | + | + |
| Croatia | Zrmanja, Krka | + | + |
| Finland | lakes Änattijärvi, Lentiira, Lammasjärvi and Saunajärvi | + | – |
| Finland | river Korojoki and lakes Auttijärvi, Koppelojärvi and Kostonjärvi, Kaukuanjärvi | + | – |
| France | Erdre, Morbihan, Vilaine and tributaries: Ille, Meu, Seiche, Semnon, Chère, Canut | + | + |
| France | Eyre, lower Charente | + | + |
| France | Leyre | + | + |
| France | Lez, Salat | + | + |
| France | middle and lower Rhône tributaries: Véore, Eyrieux, Drôme, Roubion, upper Ardèche, Gard, Buëch, upper Durance | + | partly |
| France | middle Dordogne, Céou | + | + |
| France | NW Corsica | + | + |
| France | Saison, Aspe, Ossau, Oloron, Pau | + | + |
| France | upper Garonne tributaries: Hers, Tarn with Rance, Dourdou, Dourbie, lower Aveyron with Lére and Cérou, Lémance, Ruisseau de Vert | + | + |
| France | Var and tributaries: Tinée, Vésubie, Esteron | + | partly |
| France, Spain | Agly, Aude with Fresquel, Orbieu; Orb; Hérault with Vis; Lez; Vidourle | + | + |
| France, Switzerland | Saône tributaries: Doubs, Dessoubre, Allaine, Loue | + | partly |
| Germany | Havel, Uecker | + | + |
| Germany | Lake Ammer | + | – |
| Germany | Ruhr with tributary Möhne | + | + |
| Germany | upper Danube tributaries: lower Blau, Günz, Mindel, Brenz, Egau, Zusam | + | partly |
| Germany | upper Neckar and tributaries: Fils, Rems, Murr, Enz | + | partly |
| Germany, Austria | Tiroler Achen, Lake Chiem, Alz | + | + |
| Germany, Switzerland, Austria | Lake Constance | + | + |
| Greece | Athens area | + | + |
| Greece, Turkey | Evros, Ergene | + | + |
| Greece | Aliakmonas, Loudias, Lake Orestiada (Kastoria) | + | + |
| Greece | Pinios, Ladonas, Alfeios, Evrotas, Kifisos, Spercheios, middle Pineios | + | + |
| Greece | Lake Volvi, Angitis, Kompsatos, Lake Vistonida | + | partly |
| Greece | Corfu, Kefallonia, Lesbos, Euboea, SW Crete, Chios, Rhodes, Karpathos | + | + |
| Greece | Kalamas/Thyamis, Acherontas, Louros, Arachthos, upper and lower Acheloos | + | partly |
| Greece, North Macedonia | Lake Dojran | + | + |
| Hungary | Ikva, Répce | + | + |
| Hungary | Pécsi-víz, Gyöngyös | + | + |
| Hungary | Vidi-ér, Keleti-főcsatorna, Kutas, Ölyvös, Konyári-Kálló | + | + |
| Hungary, Austria | Mosoni Duna | + | + |
| Iceland | Lake Thingvalla | + | + |
| Ireland | Iveragh peninsula, Lake Leane | + | x |
| Ireland, Northern Ireland | Lake Melvin | + | – |
| Italy | Anapo, Cassibile, Dirillo, Maroglio (Sicily) | + | + |
| Italy | Arno, Cornia, Bruna, Mignone, Paglia, Chiascio, Topino, Teverone, Lake Trasimeno | + | partly |
| Italy | Canale dei Regi Lagni | + | + |
| Italy | Cedrino (Sardinia) | + | + |
| Italy | Isola d'Elba | + | + |
| Italy | Lake Garda | + | + |
| Italy | Ligurian coast, Magra | + | + |
| Italy | Po tributaries: Orco, Stura di Lanzo, Cervo | + | partly |
| Italy | Salento peninsula | + | partly |
| Italy | Torre, Natisone | + | + |
| Italy | upper Liri, upper Salto | + | + |
| Malta | Malta | + | + |
| Montenegro | Komarnica | + | + |
| Montenegro | Moraca, Bojana | + | partly |
| Montenegro, Albania | Lake Skadar | + | + |
| Portugal | lower Mondego, Ceira, Alva; da Tornada, Lisandro | + | + |
| Portugal | Mira, Odelouca, Arade, Quartieria, Terges | + | partly |
| Romania | Lăpuş | + | + |
| Romania | lower Danube below Iron Gate and tributaries e.g. Topolniţa, Desnăţui, lower Jiu, lower Olt, Călmăţui, Lake Suhaia, lower Vedea | + | – |
| Romania | upper Argeş | + | + |
| Romania, Hungary | Barcau, Ier | + | + |
| Romania, Hungary | Crişul Negru | + | + |
| Romania, Ukraine, Moldava | Danube delta | + | – |
| Russia | Lake Kezenoyam | + | + |
| Russia | Lake Ladoga | + | – |
| Russia | lower Don | + | – |
| Russia | lower Terek & Lower Sulak | + | – |
| Russia | middle Ural, Chagan | + | – |
| Russia | Volga below Saratov, Volga delta | + | partly |
| Serbia, Bulgaria | lower Timok, lower Bedem | + | – |
| Slovakia, Hungary | Ipel' | + | – |
| Slovenia | middle Sava, Savinja with Hudinja, lower Mirna, Krka | + | + |
| Slovenia | Reka | + | + |
| Slovenia, Bosnia & Herzegovina | Ljubljanica, Ižica | + | + |
| Slovenia, Italy | Rizana | + | + |
| Spain | Embalse de Valparaíso | - | + |
| Spain | Francoli, Belcaire, Palancia, lower Turia, Júcar, Serpis | + | + |
| Spain | Genil, Blánco, Cabra, Anzur, Cacín | + | + |
| Spain | Guadalbullón, Rumblar, Jándula, Yeguas | + | partly |
| Spain | Guadalfeo, Izbor | + | + |
| Spain | Guadalquivir delta (Doñana), Guadalete, Barbate, Guadiaro, Guadalevín | + | partly |
| Spain | Guadazaón | + | + |
| Spain | Mallorca, Menorca | + | + |
| Spain | Muga | + | + |
| Spain | Narcea, Pigüeña, Caudal, Pisueña, Asón, Deba, Urumea | + | + (+ Nalón) |
| Spain | Oca, Arga, Jalón | + | + |
| Spain | upper Guadalquivir and tributaries: Guardal, Baza, Castril, upper Guadalimar | + | + |
| Spain, Portugal | Chanza, Malagón | + | – |
| Spain, Portugal | Rio Águeda | + | – |
| Switzerland | Lake Lucerne | + | + |
| Switzerland | Lake Neuchâtel | + | + |
| Switzerland | Lake Thun | + | + |
| Switzerland, France | Lake Geneva | + | + |
| Ukraine | Dniester estuary | + | – |
| Ukraine | lower Dnieper, lower Inhulets | + | – |
| Ukraine | Southern Bug estuary | + | – |
| Ukraine/Russia | SE Crimea, W Black Sea coast of Caucasus | + | + |
| United Kingdom | Eachaig with Lake Eck; Lake Ericht; Isle of Skye catchments | + | + |
| United Kingdom | Eden, Derwent, Lake Windermere | + | + |
| United Kingdom | Lake Neagh (N Ireland) | + | – |
| United Kingdom | Lower Lake Erne | - | + |
| United Kingdom | NW Scotland coastal catchments | + | + |
| United Kingdom | Shetland | + | + |
| United Kingdom | Snowdonia, Lleyn, Conwy, Dee, Mawddach, Anglesey island | + | + |
| United Kingdom | Stinchair with Duisk, coastal catchments in W Galloway, S Ayrshire | + | + |

**Part 1. Determining an optimal value for the Boundary Length Modifier (BLM)**

In Marxan, the parameter Boundary Length Modifier (BLM) controls the length of the boundaries of the optimal network relative to the area selected for protection. Higher BLM forces the solution to be more clumped, leading to more aggregated networks that may or may not be easier or less costly to effectively protect than more fragmented networks which often result when lower BLM values are specified by the user. To find an optimal value for the BLM parameter, we ran the most comprehensive no lock-in scenario by varying the BLM at eight levels (0.01, 0.1, 1.0, 10, 25, 50, 75, 100). We compared the resulting networks based on (i) the total length of the boundaries relative to the area protected as recommended in **Stewart & Possingham (2005)[[1]](#footnote-2)**, (ii) the balance of the representation of different parts of Europe in the best protected area network, and (iii) the coverage of species of conservation concern by the resulting protected area networks.

The results showed that total boundary length decreased with total area above BLM = 0.1, as expected (**Fig. S1**). The maximum cost specified (17% of total area to be protected) was exceeded between BLM 10 and 25 and total boundary length did not change much relative to total area protected above BLM 25 (**Fig. S1**). A comparison of the best protected area networks at the eight BLM values showed that the network became more compact with increasing BLM as expected (**Fig. S2**). However, catchments in southern Europe also became more emphasised compared to catchments in northern Europe with increasing BLM, and one of the greatest jumps in this tendency was between BLM 10 and 25 (**Fig. S2**). Finally, the number of threatened species not covered adequately (i.e., the specified targets regarding the proportion of range protected was not met) by the best network decreased with increasing BLM, with the largest drop occurring between BLM 1 and 10 (**Fig. S3**). Based on these considerations, BLM = 10 appeared to be a suitable compromise between fragmentation/aggregation, representation of northern and southern catchments and coverage of species in the protected area network.

Our results showed that by selecting BLM = 10 we avoided solutions that were too fragmented and placed a higher emphasis on clumping of priority catchments (see **Fig. S2**). This step ensured that if a catchment was selected in a Marxan run, neighbouring catchments were also more likely to be selected than in the case of a low BLM value. Moreover, by focusing on irreplaceability as measured by selection frequency in 1000 runs (solutions), we obtained an “average” measure of conservation priority (**Moilanen et al. 2009**)[[2]](#footnote-3). Any one solution (result of individual runs) usually returned a highly fragmented network of catchments (**Fig. S2**), but when these solutions were “averaged”, catchments along a river system clearly showed a more clumped pattern (e.g. **Fig. 1**), with higher average irreplaceability for the entire river system, and reduced risks of obtaining a highly fragmented “averaged” solution.


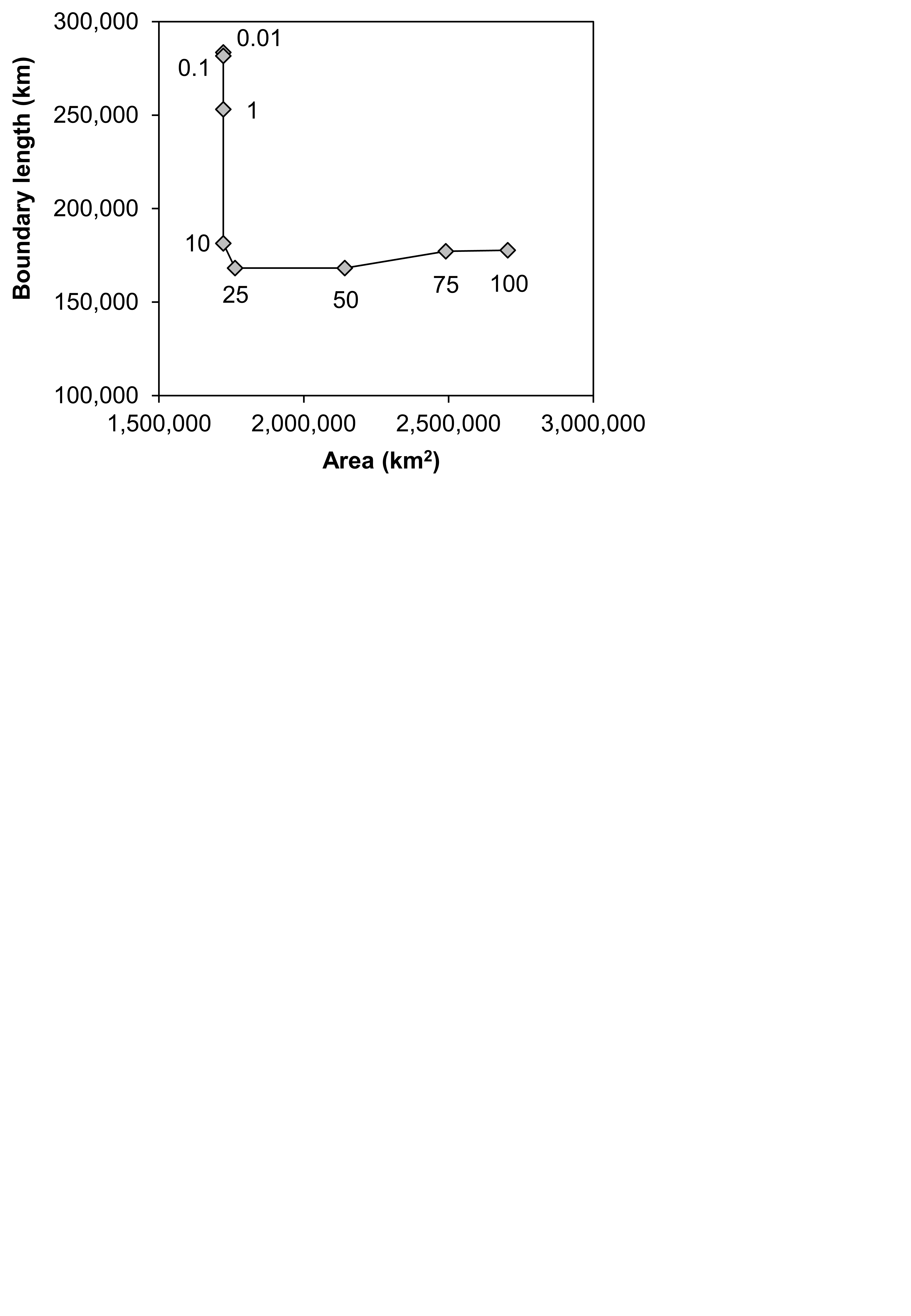


**Fig. S1.** Total boundary length as a function of total area protected in Marxan runs to determine an appropriate value for Boundary Length Modifier parameter that controls the compactness of the resulting network. BLM values are given near datapoints.

**Fig. S2.** A comparison of the best networks at eight different levels of the BLM parameter.

**Fig. S3.** The number of threatened (CR, EN, VU) and not threatened (NT, LC, DD) species that were not adequately covered by the best Marxan solution at different levels of the Boundary Length Modifier in the scenario when the proportion of protected areas was not considered in the prioritisation.

**Part 2. Determining an optimal value for the Connectivity Penalty (CP)**

In Marxan, the parameter Connectivity Penalty (CP) controls the connectivity of the optimal network relative to the area selected for protection. Higher CP values force the solution to be more connected, leading to networks containing more connected catchments, whereas lower CP values will result in networks of less connected catchments. We followed **Hermoso et al. (2011)[[3]](#footnote-4)** in creating and using the connectivity file, which specified the upstream connectivity of catchments (information available in the HydroBASINS database). We calibrated CP by running the most comprehensive no lock-in scenario by varying CP values at eight levels (0.01, 0.1, 1.0, 10, 25, 50, 75, 100) and by comparing the relationship between total boundary length and area at different levels of CP, as in **Hermoso et al. (2011)**.

The results showed that total boundary length increased with total area protected (**Fig. S4**). There was a large increase in cost (area) between CP = 10 and CP = 1.0, with a relatively smaller decrease in total boundary length. CP = 10 thus represented the last optimal value where the increase in connectivity was associated with a proportional increase in cost, whereas above CP = 10, the cost increase was higher than the relative gain in connectivity (**Fig. S4**). Based on this result, we used the value CP = 10 throughout the final Marxan runs.


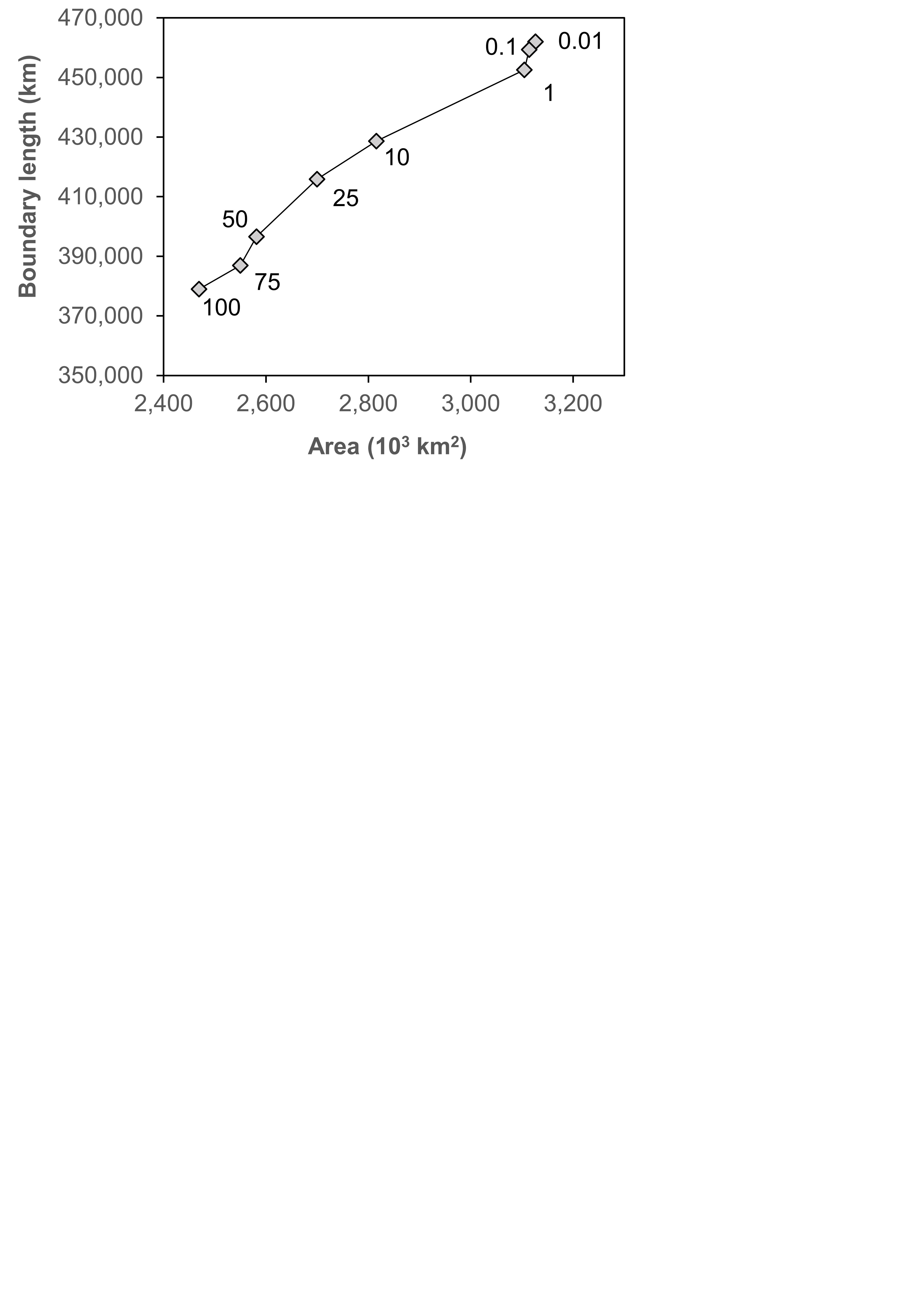


**Figure S4**. Total boundary length as a function of total area protected in Marxan runs to determine an appropriate value for the Connectivity Penalty parameter that controls the upstream connectivity of the resulting network. CP values are given near datapoints.

1. Stewart, R. R., Possingham, H. P., 2005. Efficiency, costs and trade-offs in marine reserve system design. Environmental Modelling and Assessment 10, 203-213. [↑](#footnote-ref-2)
2. Moilanen, A., Wilson, K. A., Possingham, H. P. 2009. Spatial Conservation Prioritization: Quantitative Methods and Computational Tools. Oxford University Press, Oxford. [↑](#footnote-ref-3)
3. Hermoso, V., Linke, S., Prenda, J., Possingham, H. P. 2011. Addressing longitudinal connectivity in the systematic conservation planning of fresh waters. Freshwater Biology 56, 57-70. [↑](#footnote-ref-4)
